# Role of Stereochemistry on Electron Transport in Peptides

**DOI:** 10.1021/acs.nanolett.6c04036

**Published:** 2026-10-21

**Authors:** Rajarshi Samajdar, Hemani Chhabra, Moeen Meigooni, Seungjoo Yi, Xiaolin Liu, Jason L. Wu, Emad Tajkhorshid, Charles M. Schroeder

**Affiliations:** † Department of Chemical and Biomolecular Engineering, 14589 University of Illinois Urbana−Champaign, Urbana, Illinois 61801, United States; ‡ Beckman Institute for Advanced Science and Technology, University of Illinois Urbana−Champaign, Urbana, Illinois 61801, United States; § Center for Biophysics and Quantitative Biology, University of Illinois Urbana−Champaign, Urbana, Illinois 61801, United States; ∥ Department of Materials Science and Engineering, University of Illinois Urbana−Champaign, Urbana, Illinois 61801, United States; ⊥ Department of Chemical and Biological Engineering, 6740 Princeton University, Princeton, New Jersey 08544, United States; # Department of Chemistry, Princeton University, Princeton, New Jersey 08544, United States; ∇ Department of Chemistry, University of Illinois Urbana−Champaign, Urbana, Illinois 61801, United States; ○ Department of Bioengineering, University of Illinois Urbana−Champaign, Urbana, Illinois 61801, United States

**Keywords:** molecular electronics, single-molecule experiments, peptide stereochemistry, electron transport, scanning tunneling microscope break junction (STM-BJ)

## Abstract

Stereochemistry underlies structure–function relationships across biology and materials science. Here, we investigate electron transport in oligopeptide stereoisomers using single-molecule experiments and computational modeling. Our results show that stereochemical modifications in tyrosine-based peptides lead to significant variations in molecular conductance and transport distance along the peptide backbone, associated with changes in aromatic side-chain interactions and electronic coupling. In alanine-based peptides, stereochemical modifications alter molecular conductance through secondary-structure interactions associated with β-turn conformations. Molecular dynamics simulations and quantum mechanical calculations reveal the molecular origins of these stereochemistry-dependent structural and electronic properties. Overall, this work shows that stereochemical modification of nonterminal amino acids provides a means to control electron transport through distinct mechanisms involving aromatic side-chain interactions and secondary structure. These results establish stereochemistry as a molecular design parameter for controlling electron transport in peptide-based electronic materials.

## Graphical abstract

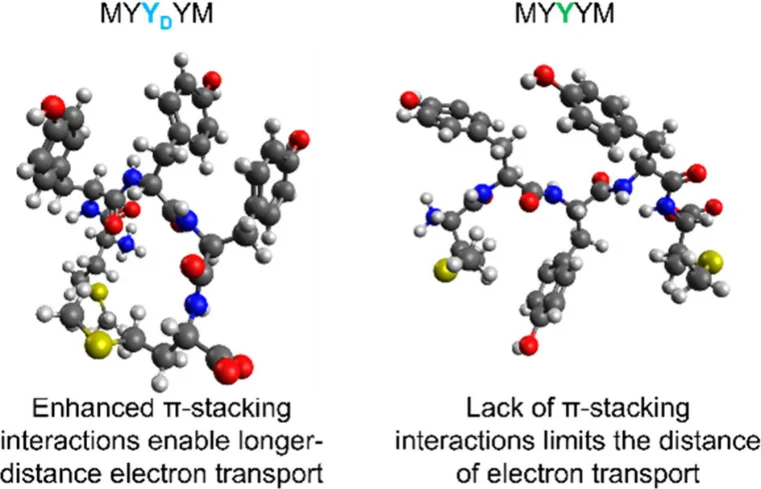

Stereochemistry governs the structure and function of synthetic and biological materials, influencing molecular conformation, intermolecular interactions, and the optical and electronic properties of a broad range of systems.^1,2^ Stereochemical effects play a central role in chemical reactions,^3^ transmission of photoexcited electrons through chiral media,^4–7^ and electron transport through chiral materials under an applied bias.^8–13^ Prior work has focused on understanding the influence of stereochemistry on electron transport in biomolecules such as DNA^8,10^ and proteins,^14^ but systematic studies that directly connect stereochemical variation to sequence-dependent conformations and transport pathways in peptides remain limited. Despite recent progress, the relationship between stereochemistry, molecular sequence and structure, and electron transport in peptides is not fully understood.

Prior work on elucidating the effect of chirality on electron transport has focused on techniques such as magnetic conductive-probe atomic force microscopy (mCP-AFM),^10^ magnetoresistance in a film,^15^ and electrochemical measurements with a ferromagnetic electrode.^16^ These experiments are typically conducted at the bulk scale or through multiple molecular layers for complex biological systems. Additional work has focused on understanding molecular-scale electron transport in peptides, revealing that stereochemical variations in an α-helical peptide sequence of 22 amino acids alter the electronic properties using asymmetric Au and Ni metal electrodes.^17^ Nevertheless, the influence of stereochemistry on the electronic properties of short peptide sequences consisting of four to five amino acids (tetra- and pentapeptides) with symmetric metal electrodes has not been fully explored. Recent work showed that electron transport in peptides shows a two-state conductance profile due to the conformational flexibility of peptide backbones, with a high-conductance state arising due to a defined secondary structure (beta turn or 3_10_ helices) and a low-conductance state arising for extended peptide structures.^18^ From this perspective, investigating electron transport in short peptide sequences with different amino acid stereoisomers is critical for revealing how stereochemistry influences the electronic properties of peptides.

Bulk scale experiments have shown that stacking of aromatic amino acids such as tyrosine lead to enhancements in electronic conductivity.^19^ Building on these observations, we conjectured that stereochemical variations in peptide sequences could modulate molecular-scale electron transport in a conformation-dependent manner. In sequences containing consecutive tyrosine residues, replacing an internal l-tyrosine with d-tyrosine is predicted to yield structures with closer spatial alignment of adjacent aromatic side chains (Scheme 1a), thereby altering π-stacking interactions and electronic coupling and potentially extending the transport pathway along the peptide backbone. Conversely, in alanine-based sequences, replacing an internal l-alanine with d-alanine is thought to promote β-turn conformations in short peptides (Scheme 1b),^20,21^ thereby potentially enhancing electron transport through secondary structure-mediated pathways. In this way, we hypothesized that stereochemical modifications in tyrosine-based peptides would influence electron transport primarily along the molecular backbone, whereas analogous modifications in alanine-based sequences could modulate transport though intramolecular hydrogen-bonded pathways.

**Scheme 1:**
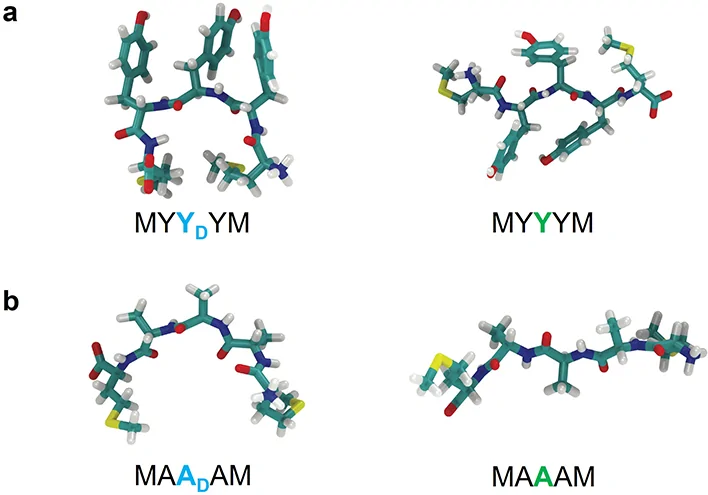
Understanding the Role of Stereochemistry on Electron Transport in Peptides^a^

a (a) Substituting internal l-tyrosine with d-tyrosine residues is predicted to yield a closer spatial alignment of aromatic side chains, providing a defined electronic pathway along the peptide backbone. (b) Substituting internal l-alanine with d-alanine is predicted to favor a β-turn conformations,^20,21^ leading to a more pronounced electronic pathway due to secondary structure interactions. M, Y, and A denote the l-amino acids methionine, tyrosine, and alanine, respectively, whereas Y_D_ and A_D_ denote the corresponding d-amino acids.

In this work, we investigate the role of stereochemistry on the electronic properties of short peptides using a combination of single molecule experiments, bulk scale spectroscopic characterization, molecular dynamics (MD) simulations, and quantum mechanical (QM) calculations. A scanning tunneling microscope-break junction (STM-BJ) technique^18,22–26^ is used to experimentally characterize the molecular charge transport behavior of oligopeptides defined by different nonterminal amino acid chiral isomers. Results from single-molecule electronics experiments show that alternating l- and d-residues modulate electron transport in a sequence-dependent manner, producing a lower-conductance state that persists over a larger junction separation in tyrosine-based peptides and a more pronounced high-conductance pathway in alanine-based peptides. All-atom MD simulations demonstrate that heterochiral peptides, in which nonterminal amino acids have alternating chirality, exhibit enhanced pi-stacking interactions between the aromatic side chains of tyrosine residues. On the other hand, heterochiral pentapeptides based on alanine display pronounced secondary structure interactions due to a favorable β-turn conformation. QM calculations are further used to validate experimental results for molecular-scale electron transport. Results from single-molecule experiments on tetrapeptides show a similar yet more subtle role of stereochemical modifications on conductivity of tetrapeptides. Overall, our work illustrates that changes in stereochemistry directly affect electron transport in peptides via primary or secondary structure-mediated pathways.

Tetra- and pentapeptides were designed with nonpolar aliphatic groups and aromatic R groups, featuring variations in the stereochemistry of the chiral centers on the alpha-carbons of nonterminal amino acids (Figure 1a,b and Supplementary Figures 1–10). The chemical library consists of homochiral peptides (nonterminal amino acids with identical stereochemistry) and heterochiral peptides (nonterminal amino acids with alternating stereochemistry) based on tyrosine (Figure 1a) and alanine (Figure 1b). We began by characterizing peptides using UV–visible and fluorescence spectroscopy. Tyrosine-based peptides exhibit a distinct absorption peak at 275 nm with shoulders at 282 nm in the UV–visible spectrum (Supplementary Figure 11), along with an emission peak near 305 nm in the fluorescence spectrum (Supplementary Figure 12), consistent with the characteristic optical features of aromatic residues for peptides reported in prior literature.^27,28^ In contrast, alanine-based peptides exhibit no distinct peaks in the aromatic region (Supplementary Figures 13,14) with an absorption around 190–220 nm due to the C=O transitions of the peptide backbone.^29,30^

**Figure 1:**
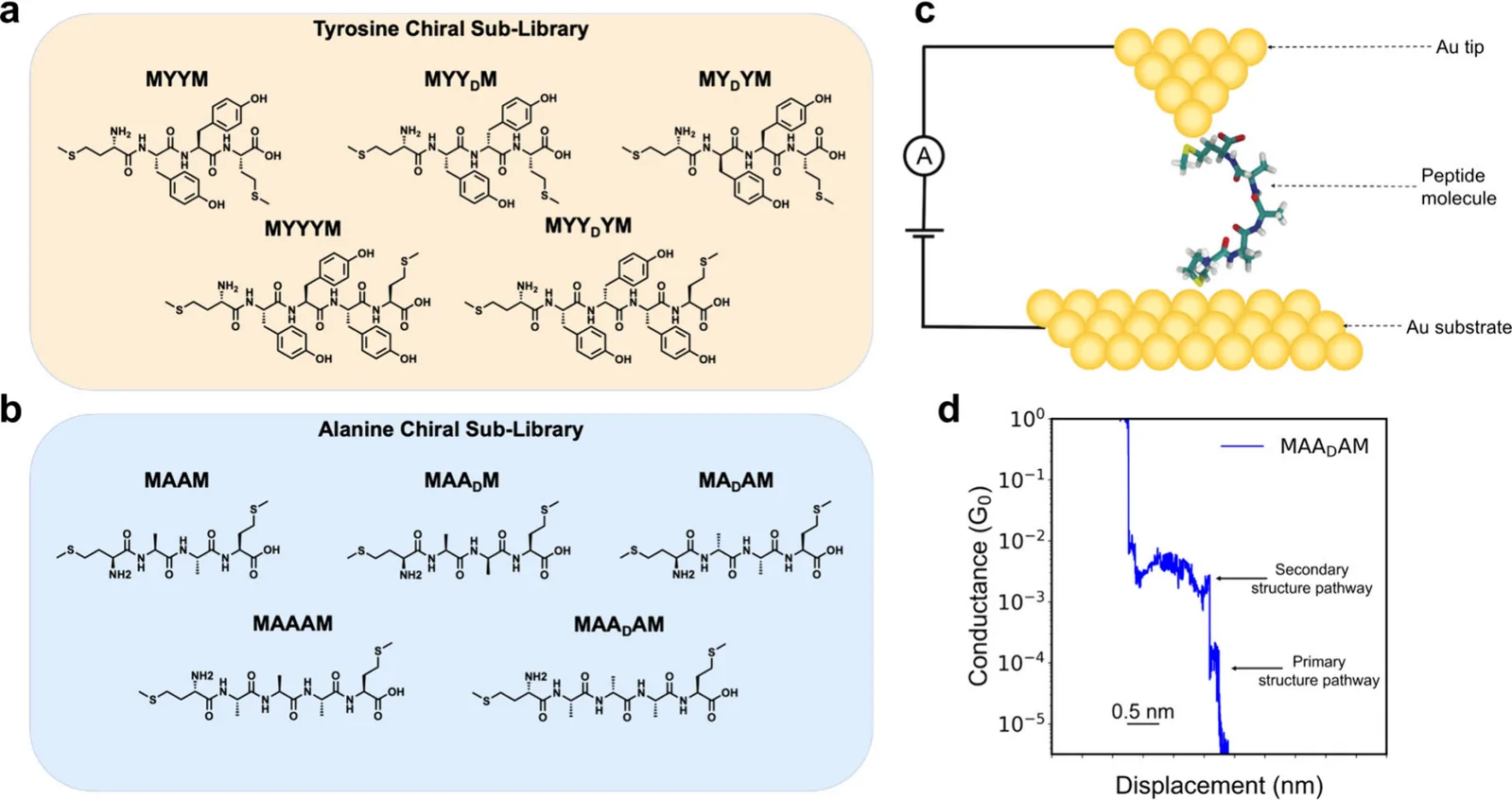
Molecular library of chiral peptides studied in this work. (a) Tyrosine-based chiral peptide sublibrary. (b) Alanine-based chiral peptide sublibrary. (c) Schematic of scanning tunneling microscope-break junction (STM-BJ) experiment with the peptide MAA_D_AM. (d) Characteristic single-molecule conductance trace for peptide MAA_D_AM, indicating a high-conductance pathway and a low-conductance pathway arising from secondary and primary structures, respectively.^18^ M, Y, and A denote the l-amino acids methionine, tyrosine, and alanine, respectively, whereas Y_D_ and A_D_ denote the corresponding d-amino acids.

To further understand the role of stereochemistry on molecular conformations, we performed two-dimensional (2D) nuclear magnetic resonance (NMR) spectroscopy. Diffusion-ordered spectroscopy (DOSY) NMR is used to determine diffusion coefficients, providing information on molecular size and shape in solution.^31,32^ We performed DOSY NMR experiments on tyrosine-based peptide sequences, as differences in these peptides could arise due to interactions between aromatic side chains (Scheme 1a). Results from DOSY NMR experiments on peptides MYYYM and MYY_D_YM revealed similar diffusion coefficients, indicating comparable molecular sizes and shapes in solution (Supplementary Figures 15,16). Nuclear Overhauser effect spectroscopy (NOESY) NMR was also used to probe through-space interactions between protons, revealing local conformational differences induced by stereochemistry.^31,33^ NOESY NMR were performed on alanine-based peptide sequences, as differences in tertiary conformation could arise due to distinct folded structures of these peptide isomers (Scheme 1b). NOESY NMR results for MAAAM and MAA_D_AM showed no observable cross-peaks (Supplementary Figures 17), suggesting the absence of close proton–proton spatial contacts in solution phase. However, methods such as NMR reflect ensemble-averaged conformations over time and across the populations of molecules in solution, and may not capture transient or folded states, if they exist in only minor subpopulations. To further probe the presence of folded conformations, we performed circular dichroism (CD) spectroscopy,^18,34^ which is more sensitive to secondary structure and can detect subtle changes in backbone conformation induced by chirality.^35,36^

Circular dichroism (CD) spectra (Supplementary Figures 18–21) indicate the presence of hydrogen bonding (H-bonding) interactions for the tetra- and pentapeptides, showing spectral features expected for beta turn or 3_10_ helices, such as a maximum or minimum around 200–210 nm and a shoulder or small peak around 220 nm.^37,38^ CD spectral features for 3_10_ helices are qualitatively different than the spectral features observed for alpha helices, beta sheets, or random coils.^39^ Additionally, pronounced differences are observed between homochiral and heterochiral peptide sequences. Several heterochiral peptides, including MYY_D_YM, MA_D_AM, and MAA_D_AM, display spectral features in the 190–200 nm region (Supplementary Figures 19–21) that differ from their homochiral counterparts. Furthermore, the CD spectrum of MY_D_YM appears as a shifted mirror image of that of MYYM (Supplementary Figure 18), highlighting the role of amino acid stereochemistry on peptide conformation. These observations indicate that peptides composed of the same amino acid sequence but differing in the stereochemistry of nonterminal residues adopt markedly different conformational ensembles. Such differences likely arise from changes in side-chain orientation, intramolecular interactions, and three-dimensional conformational arrangements induced by stereochemical variation. We next performed single-molecule measurements to understand how peptide structural variations affect electron transport.

Tetra- and pentapeptides were designed with a methionine residue at the N- and C-termini. Methionine contains a methyl sulfide (-S-CH_3_) group that readily binds to gold,^40^ thereby providing electrical contacts to metal electrodes in the STM-BJ setup (Figure 1c). Although neutral amines are known to bind to Au in STM-BJ measurements,^41,42^ the peptides studied here are characterized in water (peptide concentration <1 mM) at pH 7, where the free N- and C-termini are expected to exist predominantly in their zwitterionic forms (NH_3_^+^ and COO^–^, respectively). Thus, the N-terminal amino group does not provide the same neutral lone-pair donor motif required for canonical amine–Au coordination. Prior STM-BJ studies on peptides have shown that molecular junctions formed through native peptide termini (NH_3_^+^ and COO^–^)^43^ are electronically distinct from those formed using stronger covalent or sulfur-based anchors.^18,44^ Prior work^18^ revealed a two-state molecular conductance behavior of homochiral peptides due to the conformational flexibility of peptide backbones, with a high-conductance state arising due to secondary structure interactions (beta turn or 3_10_ helices) and a low-conductance state occurring for extended peptide structures with the terminal methionine groups serving as the predominant Au-binding motifs (Figure 1d). Gaussian mixture modeling (GMM)-based unsupervised learning further supported this assignment by linking distinct conductance features to conformation-mediated electron transport through peptide backbones (Supplementary Figures 22–25). Importantly, the high- and low-conductance populations occur together within the same dominant cluster of molecular traces, supporting their assignment to dynamically interconverting peptide configurations rather than distinct conductance populations arising primarily from different anchoring motifs. While this analysis does not definitively exclude contributions from alternative terminal interactions, including interactions involving the N-terminal amino group, the collective results support a predominantly conformation-dependent origin of the two conductance states.

Although saturated SMe–alkane–SMe junctions^45^ provide useful benchmarks for nonconjugated molecular wires, peptide backbones are structurally distinct because intramolecular hydrogen-bonding interactions can create folded transport pathways with enhanced electronic coupling relative to extended alkane chains.^18^ Across all peptide sequences studied in this work, the conductance histograms display a well-defined, reproducible Au–Au point-contact peak, essentially coincident with the conductance quantum *G*_*0*_, confirming reliable contact formation and conductance calibration throughout the data set (Supplementary Figure 26). Solvent-only STM-BJ controls show Au–Au rupture followed by tunneling decay, without molecular features corresponding to molecular conductance signatures (Supplementary Figure 27). In addition, control experiments with Gly-Gly-Gly-Gly reproduce the weak conductance behavior reported previously for unmodified oligopeptides^43^ (Supplementary Figure 28). The single-molecule conductance data sets for the homochiral reference peptides MYYM, MYYYM, MAAM, and MAAAM were previously reported in our earlier study^18^ and are included here for direct comparison with the corresponding modified peptide sequences. The present work aims to understand how changes in stereochemistry affect the molecular electronic properties of peptides.

We began by characterizing the electron transport behavior of tyrosine-based tetrapeptides. Our results show that the high-conductance state of tyrosine-based homochiral tetrapeptides (MYYM) is similar to the high-conductance state of heterochiral tetrapeptides (MYY_D_M and MY_D_YM) around ∼10^–2.8^ *G*/*G*_0_, suggesting that the electron transport pathway mediated by secondary structure interactions is not significantly altered by stereochemical changes in tyrosine-based sequences (Supplementary Figure 29 and Supplementary Table S1). In contrast, the low-conductance state, corresponding to the electron transport pathway along the primary peptide backbone, shows significant variation between chiral isomers in tyrosine-based peptides, with the heterochiral sequence MYY_D_M exhibiting a conductance value ∼10^0.5^ *G*/*G*_0_ smaller than the homochiral peptide MYYM (Supplementary Figure 29 and Supplementary Table S1).

The effect of stereochemistry on molecular conductance was found to be more pronounced in tyrosine-based pentapeptides (Figure 2). Characteristic single-molecule traces (Figure 2a) show that stereochemical changes in tyrosine-based pentapeptides result in a pronounced low conductance feature. One-dimensional and two-dimensional molecular conductance histograms generated for ensembles of >5000 single molecules (Figure 2b,c,d) reveal that the low conductance state is affected by stereochemical changes in tyrosine, with the peak molecular conductance value for MYY_D_YM around ∼10^–4.8^ *G*/*G*_0_ compared to the peak molecular conductance of MYYYM around ∼10^–4.0^ *G*/*G*_0_ (Supplementary Table S2). Notably, although MYY_D_YM exhibits a lower conductance magnitude than MYYYM, its molecular conductance feature persists over a significantly larger junction separation, indicating that stereochemical modification extends the distance over which electron transport occurs while altering electronic coupling along the peptide backbone. Based on these results, we posit that alternating stereochemistry of internal amino acid residues in tyrosine-based sequences results in enhanced spatial alignment of aromatic side chains, thereby promoting pi-stacking interactions, enhancing electronic coupling, and creating a defined electron transport pathway along the primary backbone. This hypothesis is evaluated using molecular simulations and modeling, as discussed below.

**Figure 2:**
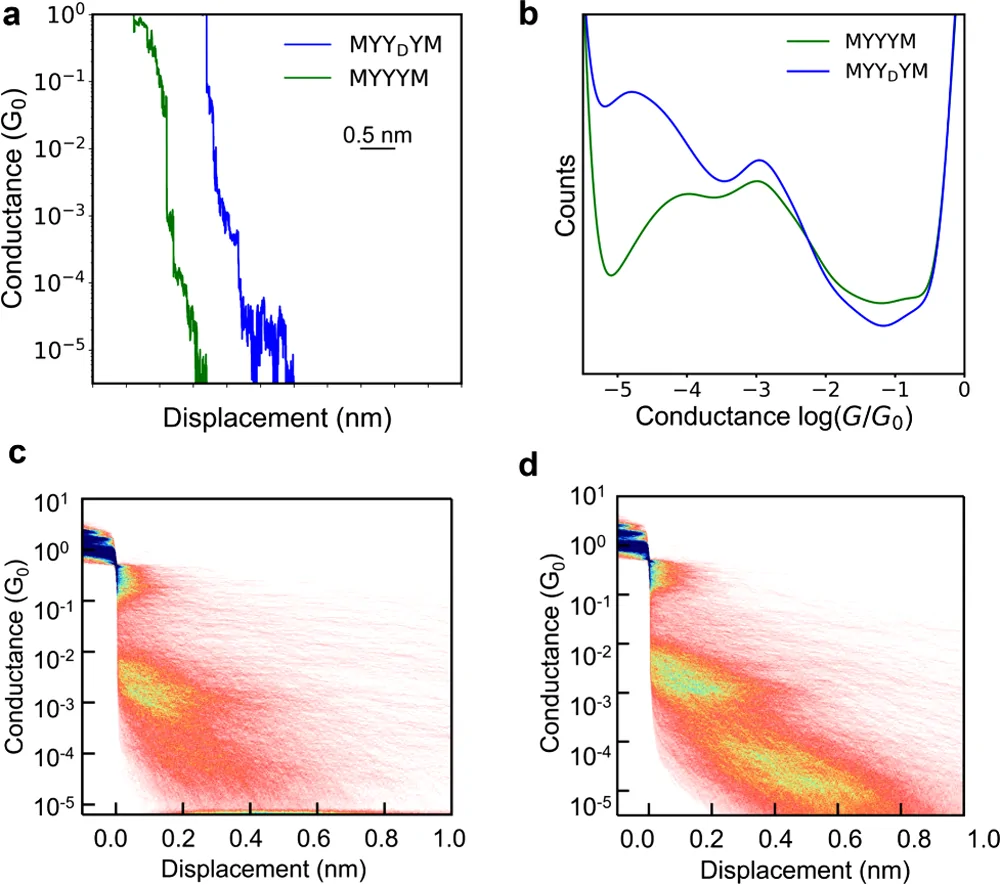
Single molecule electronic measurements for tyrosine-based pentapeptides. (a) Characteristic single molecule traces for MYYYM and MYY_D_YM. (b) 1D conductance histograms for homochiral peptide MYYYM and heterochiral peptide MYY_D_YM. 2D conductance histograms for (c) MYYYM and (d) MYY_D_YM. M, Y, and A denote the l-amino acids methionine, tyrosine, and alanine, respectively, whereas Y_D_ and A_D_ denote the corresponding d-amino acids. All data were obtained using 0.1 mM concentrations of peptoids in water at 250 mV applied bias across ensembles of at least 5000 single molecules. The MYYYM single-molecule conductance data set was previously reported in ref 18 and is replotted here for direct comparison with MYY_D_YM. Reference 18 is available under a CC BY 4.0 license. Copyright 2024.

We next performed single-molecule electronic experiments on alanine-based tetra- and pentapeptides. Our results show that alanine-based homochiral and heterochiral tetrapeptides show similar high and low conductance features (Supplementary Figure 30 and Supplementary Table S3). However, alanine-based heterochiral tetrapeptide sequences show a higher occurrence of the high conductance state compared to homochiral alanine-containing tetrapeptides (Supplementary Figure 30a). Characteristic single-molecule traces for alanine-based pentapeptides (Figure 3a) show an enhanced high-conductance feature for heterochiral peptides with alternating stereochemistry of internal amino acid residues, which likely arises due to enhanced secondary structure interactions. One-dimensional and two-dimensional molecular conductance histograms generated across ensembles of >5000 single molecules (Figure 3b,c,d and Supplementary Table S4) reveal higher counts and a longer molecular junction distance for MAA_D_AM compared to MAAAM. Alternating the chirality of alanine residues in a peptide sequence is known to promote favorable β-turn conformations,^20,21^ which could support an enhanced electronic pathway due to secondary structure interactions. To further understand these findings, computational modeling is used to rationalize the experimental results.

**Figure 3:**
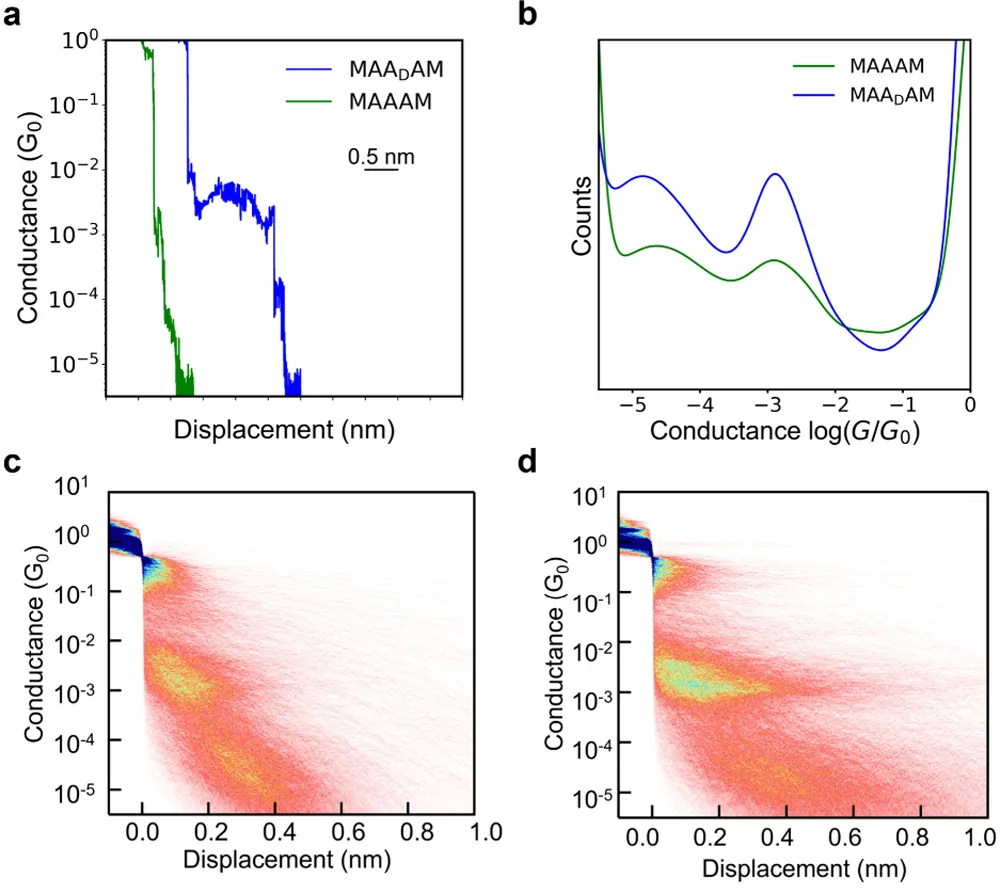
Single molecule electronic measurements for alanine-based pentapeptides. (a) Characteristic single molecule traces for MAAAM and MAA_D_AM. (b) 1D conductance histogram for MAAAM and MAA_D_AM. 2D conductance histogram for (c) MAAAM and (d) MAA_D_AM. M, Y, and A denote the l-amino acids methionine, tyrosine, and alanine, respectively, whereas Y_D_ and A_D_ denote the corresponding d-amino acids. All data were obtained using 0.1 mM concentrations of peptoids in water at 250 mV applied bias across ensembles of at least 5000 single molecules. The MAAAM single-molecule conductance data set was previously reported in ref 18 and is replotted here for direct comparison with MAADAM. Reference 18 is available under a CC BY 4.0 license. Copyright 2024.

To understand the role of molecular conformation on electron transport, we performed MD simulations for tyrosine and alanine-based peptides (Figure 4a). MD simulations were performed in explicit solvent with a series of custom potentials to implicitly represent interactions between peptides and gold electrodes, as described in prior work^18^ (Supplementary Figure 31). The projection of the end-to-end distance (sulfur anchor-to-anchor distance on terminal methionines) of the peptide along the experimental pulling axis was harmonically restrained to a series of distances (6 Å, 9 Å, and 12 Å), allowing the peptide to adopt an ensemble of conformations. MD simulations for homochiral peptides, MAAAM and MYYYM, show that 2→5 backbone hydrogen bonds (H-bonds) form with remarkable consistency during the 6 Å end-to-end holding distance, but the interactions vanish when the end-to-end distance is 12 Å (Supplementary Figure 32). This observation is consistent with our experimental findings in this work and prior work based on single-molecule experiments.^18^ We also analyzed MD simulation results to elucidate the molecular origin of the lower-conductance but longer-distance transport state observed for MYY_D_YM compared with MYYYM, presumably associated with electron transport along the primary backbone, and the origin of the pronounced high-conductance state in MAA_D_AM compared to MAAAM, presumably associated with electron transport due to secondary structure interactions.

**Figure 4:**
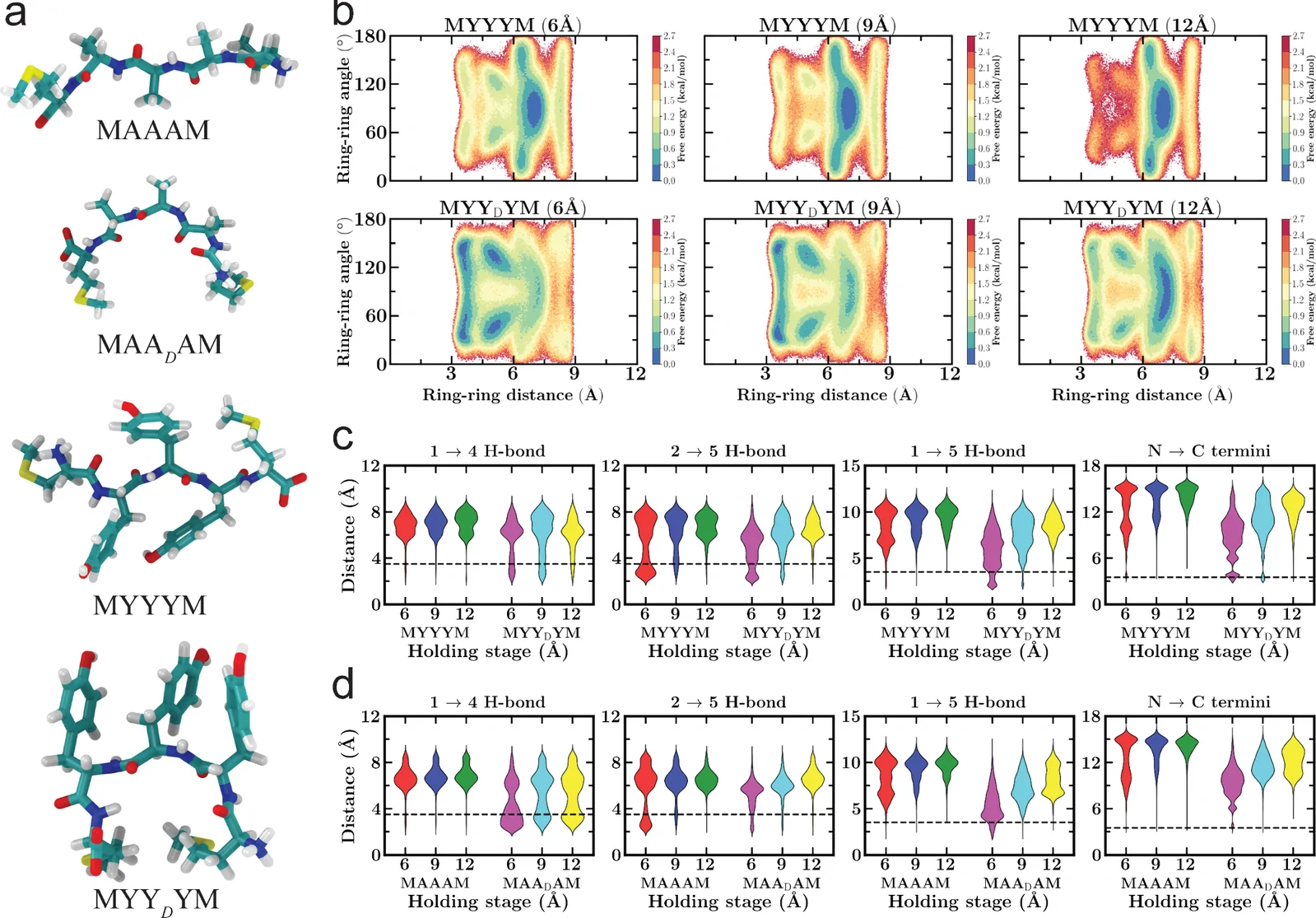
MD simulation results for homochiral and heterochiral pentapeptides. (a) Snapshots for alanine- and tyrosine-based pentapeptides studied in this work. (b) Free energy plots for tyrosine-based pentapeptides as a function of the distance and angle between the aromatic side chains, indicating increased interactions between phenyl rings in heterochiral peptides as compared to homochiral peptides. (c, d) Violin plots showing backbone H-bonding distance distribution for tyrosine and alanine based pentapeptides. M, Y, and A denote the l-amino acids methionine, tyrosine, and alanine, respectively, whereas Y_D_ and A_D_ denote the corresponding d-amino acids. A black dotted line in the violin plots indicate the cutoff for intermolecular H-bonding.

The energy profiles for tyrosine-based homochiral and heterochiral sequences as a function of the distance and angle between the aromatic side chains indicate closer spatial alignment and enhanced interactions between the phenyl rings in MYY_D_YM compared to MYYYM (Figure 4b). Violin plots of backbone H-bonding distances for tyrosine-based peptides indicate a more pronounced population at larger end-to-end distances (9 Å and 12 Å) for MYY_D_YM compared to MYYYM (Figure 4c). These results indicate that stereochemistry-dependent aromatic interactions alter the geometry of the primary-structure transport pathway and enable junction-compatible configurations at larger end-to-end distances, consistent with the increased junction displacement observed experimentally. Overall, MD simulations and analyses based on backbone H-bonding, as well as free energy landscapes describing the orientation and alignment of phenyl side chains, support the molecular and structural basis for the enhanced electronic pathway along the primary structure observed in heterochiral tyrosine-based peptides.

Violin plots for MAAAM and MAA_D_AM based on backbone H-bonding are shown in Figure 4d. Our results reveal that MAA_D_AM has a prominent population at small end-to-end distances (6 Å) compared to MAAAM due to a 1→4 H-bonding interaction, indicating increased folding (β-turn or 3_10_ helix character), whereas MAAAM predominantly exhibits a 2→5 H-bonding interaction (Figure 4d). From this perspective, these results suggest that the probability of forming secondary structures, such as 3_10_ helices, strongly influences electronic functionality depending on the conformation adopted by the peptide. For instance, a 1→4 H-bonding interaction can differ from a 2→5 H-bonding interaction. Overall, results from all-atom MD simulations suggest that the heterochiral peptide MAA_D_AM has a prominent electron transport pathway due to secondary structure interactions owing to more favorable β-turn or 3_10_ helical formations compared to the homochiral peptides, consistent with single molecule experimental results. Next, quantum mechanical calculations and nonequilibrium Green’s function–density functional theory (NEGF-DFT) simulations were used to further to rationalize experimental results.

To complement the single-molecule conductance measurements and MD simulations, density functional theory (DFT) calculations were performed to understand the role of stereochemistry on the electronic structure of tyrosine- and alanine-based peptides. These calculations were carried out on representative peptide conformations obtained from the MD simulations, corresponding to the low-conductance state of the tyrosine-based peptides and the high-conductance state of the alanine-based peptides observed in the STM-BJ experiments. Results from frontier molecular orbital analysis (Supplementary Figures 33–36) show that stereochemical alterations do not significantly affect the energy alignment of the HOMO–1, HOMO, LUMO, and LUMO+1 levels within each peptide pair (MAAAM vs MAADAM and MYYYM vs MYYDYM), as shown in Supplementary Table S5. Based on these observations, we propose that differences in electron transport between sequences of differing stereochemistry are governed by conformational variations, supported by the MD simulations, rather than significant differences in frontier orbital energies.

To further understand the role of amino acid stereochemistry on the electronic properties of peptides, we performed projected density of states (PDOS) calculations (Supplementary Figures 37–38). PDOS analysis demonstrates that stereochemical inversion redistributes orbital character near the frontier states, as evidenced by changes in the relative contributions of different atomic sites without significant shifts in energy. Overall, results from DFT modeling highlight the importance of structural and conformational effects, rather than significant changes in electronic level alignment, in governing electron transport through these peptides.

We next performed electron transmission calculations on tyrosine and alanine-based peptides. Representative molecular structures corresponding to the most probable conformations generated by MD were used in nonequilibrium Green’s function-density functional theory (NEGF-DFT) calculations to enable direct comparison between theory and experimental results. Electron transmission calculations performed on representative peptide conformations corresponding to the low-conductance state reveal a nearly 4-fold difference in transmission at the Fermi energy between MYYYM and MYY_D_YM (Supplementary Figure 39a,b), consistent with the trends observed in single-molecule break-junction experiments (Supplementary Table 6). Electron transmission calculations for the alanine-based peptides MAAAM and MAA_D_AM, corresponding to the high-conductance state, reveal comparable transmission values at the Fermi energy (Supplementary Figure 39c,d), consistent with the experimental observations (Supplementary Table S7). Taken together, results from NEGF-DFT and quantum calculations show that alternating stereochemical sequences of tyrosine and alanine-based amino acids modulate electron transport through conformationally dependent changes in electronic coupling, rather than through shifts in frontier orbital energies.

In summary, this work provides a comprehensive experimental and computational characterization of how non-terminal amino acid stereochemistry dictates electron transport in oligopeptides. Results from single-molecule experiments and computational modeling reveal that stereochemical modifications in tyrosine-based sequences lead to significant variations in electronic conductivity along the primary structure due to increased interactions among the aromatic side chains of tyrosine residues. Moreover, chirality changes in alanine-based pentapeptides result in significant variations in electronic conductivity along the secondary structure due to more favorable β-turn conformations. All-atom MD simulations are used to analyze backbone H-bonding interactions and free energy landscapes describing the orientation and alignment of phenyl side chains, thereby providing a rational basis for understanding the putative pathways for electron transport observed in the experimental results. Quantum mechanical calculations, including NEGF-DFT, further establish a direct connection between structure, electronic structure, and transport behavior.

Overall, our work demonstrates the tuning of electronic properties at the molecular scale by varying the stereochemistry of specific amino acids within a peptide backbone, extending the junction distance over which electron transport persists through primary- or secondary-structure-mediated pathways. The insights gained from this work provide a framework for understanding how stereochemistry influences electron transport in biomolecular systems and offer design principles for engineering longer peptide and protein-inspired sequences with tailored electronic properties. By strategically varying the stereochemistry of individual amino acids, it may be possible to modulate peptide conformation, self-assembly behavior, intermolecular interactions, and charge-transport pathways, thereby enabling systematic control over electronic currents and functionality in bioelectronic materials. Moving forward, extending this approach to longer and more complex sequences, incorporating environmental effects such as solvent^26^ will be essential to fully elucidate the interplay between structure and electronic function. Broadly, these insights could be used to open new avenues for the design of bioinspired electronic materials and molecular devices, where stereochemistry can be leveraged to engineer tunable and directional charge transport pathways.

## Supplementary Material



## Notes

Methods, mass spectrometry data, UV–visible and fluorescence spectroscopy data, two-dimensional (2D) nuclear magnetic resonance (NMR) spectroscopy, circular dichroism (CD) spectroscopy data, quantum mechanical calculations, and additional single molecule electron transport experiments (PDF)
